# Bcl-2 modified adipose-derived stem cells improve the retention of fat graft

**DOI:** 10.1080/21623945.2022.2107195

**Published:** 2022-08-17

**Authors:** Ziwei Cui, Qian Tan

**Affiliations:** a Department of Aesthetic Surgery, the Daqing Oilfield General Hospital, Daqing, Heilongjiang, China; b Department of Burns and Plastic Surgery, The Drum Tower Clinical Medical College, Nanjing Medical University, Nanjing, Jiangsu, China; c Department of Burns and Plastic Surgery, Affiliated Drum Tower Hospital, Nanjing University Medical School, Nanjing, Jiangsu, China

**Keywords:** Adipose-derived stem cell, Bcl-2, gene transduction, fat transplantation

## Abstract

In cell-assisted lipotransfer, adipose-derived stem cells play a crucial role in enhancing fat graft retention. *In vitro*, human adipose-derived stem cells were modified with Bcl-2 gene. In vivo, aspirated fat was mixed with the Bcl-2-modified adipose-derived stem cells and then transplanted subcutaneously into nude mice. The retention of fat graft was evaluated. The surviving Bcl-2-modified adipose-derived stem cells were tracked after transplantation. Capillary density was quantified after transplantation. Transplantation with Bcl-2-modified adipose-derived stem cells enhanced fat graft retention by 49% and 114% at 6 weeks compared with the Fat + vector-modified adipose-derived stem cell group and Fat-only group, respectively. Transplants from the Fat + Bcl-2-modified adipose-derived stem cell group had significantly more intact adipocytes and lower levels of fat necrosis and fibrosis at 6 weeks. The survival of Bcl-2-modified adipose-derived stem cells increased by 33% at 3 weeks and 54% at 6 weeks, respectively, compared with vector-modified adipose-derived stem cells. The capillary density was 24% higher in Fat + Bcl-2-modified adipose-derived stem cell group than in Fat + vector-modified adipose-derived stem cell group or 60% higher than in Fat-only group at 3 weeks.

## Introduction

Autologous fat transplantation is a popular option for soft tissue augmentation because the technique is considered to be easily manipulated and adipose tissue can be harvested in significant amounts with less donor site morbidity and can avoid self-immune reactions and ethics concerns [1–3]. However, there are admitted defects in the therapy, such as unpredictable results and a low rate of graft retention due to partial absorption and necrosis. To improve the retention of human autologous fat grafts, some techniques associated with fat transplantation have been modified successively in the past years [4–7], these modifications focused on the techniques and instrument improvements in harvesting and processing of adipose tissue, the volume of fat implantation, sites to be implanted, and the level of placement. There is no doubt that further improvements are needed. In recent years, there has come a new technique called cell-assisted lipotransfer, as a novel cell-based strategy providing a new direction for autologous fat transfer [8–10]. In cell-assisted lipotransfer, relatively adipose-derived stem cell-poor fat is converted into adipose-derived stem cell-rich fat by adding adipose-derived stem cells to fat graft. Adipose-derived stem cells play foremost important role in enhancing fat graft retention and reducing undesirable side effects [11–14]. However, some previous studies have demonstrated that transplanted stem cells do not survive well in ischaemic conditions [15–17]. Thus, how to enhance adipose-derived stem cell survival after transplantation is crucial in cell-assisted lipotransfer.

Bcl-2-family proteins regulate all major types of cell death, including apoptosis, necrosis and autophagy. This gene encodes an integral outer mitochondrial membrane protein that blocks cell death [18,19]. As an antiapoptotic member of the Bcl-2 family, Bcl-2 serves as a critical role in regulating cell apoptosis, inhibiting cell death [20,21]. A previous study demonstrated that overexpression of Bcl-2 significantly reduces adipose-derived stem cell apoptosis in vitro [22].

In this study, we hypothesized that adipose-derived stem cells modified with Bcl-2 would achieve relatively high resistance to ischaemic conditions in vivo, and the survival number of adipose-derived stem cells would increase significantly in the early post-transplanted period. We determined whether cotransplantation of Bcl-2-modified adipose-derived stem cells and fat tissues would improve the retention of fat grafts.

## Materials and methods

### Sources of human fat tissues

The present study was approved by, and performed in accordance with, the guidelines and study protocols of the Nanjing Medical University Medical Science Research Ethics Committee. To isolate human adipose-derived stem cells, human subcutaneous adipose tissue samples were obtained from three healthy, female donors with a mean body mass index (BMI) of 25.1 undergoing abdomen liposuction. The adipose aspirate for fat transplantation was harvested from a 28-y-old healthy female patient donor with a BMI of 24.3 undergoing thigh liposuction. Before suctioning, the donor site was infiltrated with the tumescent solution containing 0.08% lidocaine and 1:500,000 of epinephrine. Adipose tissue was suctioned using a 2.5 mm inner diameter cannula connected with a 50 ml syringe in an appropriate (26–40 Kpa) negative pressure.

### Isolation and culture of adipose-derived stem cells

Adipose-derived stem cells were isolated from the fatty portion of liposuction aspirates. The aspirated fat was digested on a shaker under gentle agitation at 37°C in PBS containing 0.1% collagenase type I(Gibco, Carlsbad, Calif.) for 1 h. Mature adipocytes and connective tissues were separated from the stromal cell fraction by centrifugation. The cells were resuspended with Dulbecco’s modified Eagle medium containing 10% foetal bovine serum (Gibco). The cells were filtered through a 200-μm mesh and then centrifuged. The isolated cells were resuspended, seeded into 25-cm^2^ flasks and cultured at 37°C in a humidified atmosphere with 5% CO_2_. The medium was replaced every 3 d. Cells were passaged at a ratio of 1:3 per week. Only cells that were passaged 3 times were used in this study.

### Surface markers identification of adipose-derived stem cells

Adipose-derived stem cells were harvested by treatment with 0.25% trypsin/EDTA and then resuspended at a total number of 2 × 10^6^ cells in 500 μL PBS. The cells were incubated for 15 minutes at 4°C with the following antibodies: anti-CD29-FITC (eBioscience, San Diego, Calif.), anti-CD34-FITC (BD Biosciences, San Diego, Calif.), anti-CD44-PE (BD Biosciences), anti-CD90-FITC (BD Biosciences), anti-CD133 (293C3)-PE (Miltenyi Biotec GmbH, Bergisch Gladbach, Germany), or HLA-DR-FITC (BD Biosciences). FITC- or PE-labelled mouse IgG (Biolegend, San Diego, Calif.) was used as the isotype control. The cells were analysed with a BDFACSCanto flow cytometer (Becton-Dickinson, San Jose, Calif.). Data were analysed with the Cell Quest software package (Becton Dickinson, Bedford, Mass.).

### Genetic modification of adipose-derived stem cells

Passage 3 adipose-derived stem cells were seeded in a six-well plate at 1 × 10^5^ cells/well. At 80% confluence, the adipose-derived stem cells were infected with an adenovirus encoding green fluorescent protein (GFP) gene at different multiplicities of infection (MOI = 0, 100, 200, and 500). At 48 hours post-transduction, the transduction efficiency of the GFP gene was examined under a fluorescence microscope (ZEISS, Germany) and with flow cytometry. The optimal MOI was adopted for Bcl-2 gene delivery into adipose-derived stem cells. ADSCs without transfection and those transfected with adenovirus carrying Bcl-2 or adenovirus alone were termed ‘ADSCs’, ‘Bcl-2-ADSCs’, and ‘vector-ADSCs’, respectively. Bcl-2 protein expression levels were evaluated using Western blotting.

### Western blot analysis

The genetically modified adipose-derived stem cells were homogenized in cell lysis buffer (keygentec) and then centrifuged, and the supernatant was collected. Equal amounts of denatured protein were loaded into each lane of a gel. After electrophoresis, the resolved proteins were transferred onto a nitrocellulose membrane. The membrane was blocked in PBS buffer containing 0.2% Tween 20 and 5% skim milk for 2 hours at room temperature and then incubated with an anti-human Bcl-2 protein antibody (keygentec) overnight at 4°C. The membranes were subsequently incubated with horseradish peroxidase-conjugated secondary antibody, and immunoreactivity bands were detected by ECL kit (keygentec). Proteins collected from vector-modified adipose-derived stem cells and adipose-derived stem cells served as a control.

### Cell labelling

The cells were labelled with bromodeoxyuridine (BrdU) (Sigma-Aldrich, St. Louis.) at a final concentration of 20 µmol/l in culture medium for 48 hours and then harvested by digestion with 0.25% trypsin/EDTA. The harvested cells were resuspended in a culture medium at a density of 5 × 10^5^ per 100 µl and kept on ice until transplantation.

### Cotransplantation

All procedures were performed in accordance with the Guide for the Care and Use of Laboratory Animals (NIH publication number 85–23, revised 1996). Sixteen nude mice weighing 15–18 g served as fat transplantation models. The human aspirated fat (100 mg) was mixed with 100 µl of one of the following: 5 × 10^5^ Bcl-2-modified adipose-derived stem cells (Fat + Bcl-2-modified adipose-derived stem cell group), 5 × 10^5^ vector-modified adipose-derived stem cells (Fat + vector-modified adipose-derived stem cell group), or DMEM (Fat-only group) was subcutaneously injected into the dorsal surface of nude mice, respectively. Each mouse received all three transplants at different spots in a random fashion. Eight mice were sacrificed at 3 weeks and 6 weeks after transplantation.

### Histological analysis

At 6 weeks post-transplantation, the fat grafts were removed, weighed, and snap-frozen in liquid nitrogen. The fat grafts were embedded in the optimum cutting temperature medium (OCT) compound and cut into 6-µm-thick sections from the centre of the dissected fat grafts for haematoxylin-eosin (H&E) staining.

### Immunostaining

Frozen tissue sections were incubated with a monoclonal mouse anti-BrdU antibody (Lab Vision, Fremont, Calif.). After blocking in Envision blocking buffer, sections were placed in primary antibody overnight at 4°C to 8°C. On the following day, the sections were incubated with Alexa 568 (Molecular Probes, Eugene, OR.) conjugated goat anti-mouse IgG. Endothelial-like cells were stained for von Willebrand factor (vWF) (primary antibody: polyclonal rabbit anti-vWF antibody, H-300, Santa Cruz, Dallas, Texas; secondary antibody: Alexa 568 conjugated goat anti-rabbit IgG, Molecular Probes). Nuclei were counterstained with 4,6-diamidino-2-phenylindole (DAPI). For colocalization study, BrdU-positive cells were identified (primary antibody: monoclonal mouse anti-BrdU antibody, Lab Vision; secondary antibody: Alexa 488 conjugated goat anti-mouse IgG, Molecular Probes).

### Quantitative analysis of cell survival

Quantitative analysis of BrdU-positive cells was performed on the 6-µm-thick immunostained frozen sections at 3 weeks, and 6 weeks after transplantation. For every transplant, five slices from the centre of every dissected fat grafts were used for measurement. A total of 20 different fields of each slide were randomly selected from the region of interest and digitally photographed. Cell survival was expressed as the proportion of the BrdU-positive nuclei to the total number of nuclei. The data were analysed by two investigators in a blinded manner.

### Determination of capillary density

The capillary density in the transplanted fat tissue was determined by anti-vWF antibody staining in tissue sections at weeks 6 after transplantation. For quantification of positively stained vessels, five sections of each fat graft were analysed by a blinded reviewer. Ten different fields (200×) were randomly selected for determining the number of capillaries in two sections per fat graft. The results were expressed as capillaries per high power field (HPF).

### Statistical analysis

Data were expressed as means ± SD. Statistical analyses were performed using SPSS 16.0 software package (IBM Corp. Armonk, NY, USA). Differences among the three groups (Fat-only group, Fat + vector-modified adipose-derived stem cell group, and Fat + Bcl-2-modified adipose-derived stem cell group) were compared using one-way ANOVA. Comparisons between two groups were performed with unpaired Student’s t tests. A level of *p* < 0.05 was considered statistically significant.

## Results

### Characterization of adipose-derived stem cells

Flow cytometric analysis demonstrated that adipose-derived stem cells expressed stem cell-associated markers, CD29, CD34, CD44, and CD90, and lacked expression of the haematopoietic stem cell marker CD133 and the fibroblast marker HLA-DR.

### Genetic modification of adipose-derived stem cells

**Figure 1.**
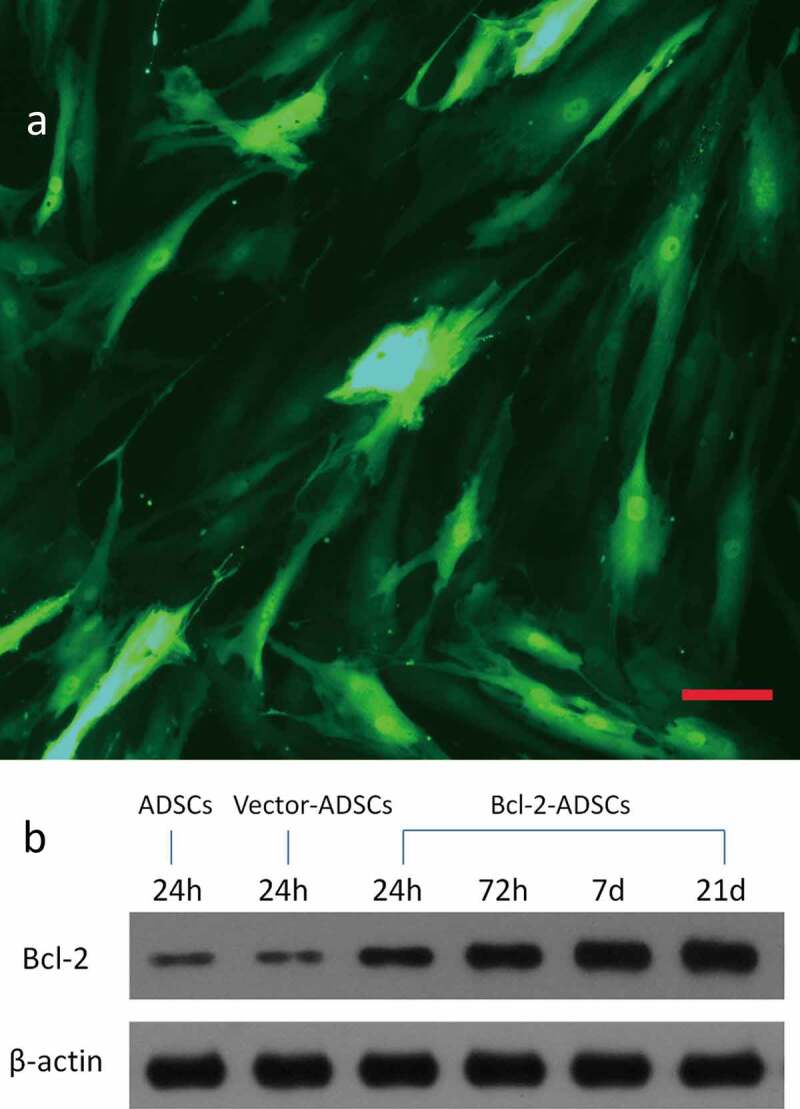
Genetic transduction of adipose-derived stem cells. (a) Representative photomicrograph of adipose-derived stem cells transducted with adenovirus encoding GFP gene. (b) Representative Western blots showing overexpression of Bcl-2 protein in Bcl-2 modified adipose-derived stem cells, which remained at a high level for 21 d after transduction. Housekeeping protein β-actin served as loading control. Scale bars = 100 μm.

At MOIs of 0, 100, 200, and 500, adenovirus encoding GFP transfection efficiencies were 0, 49%, 81.6%, and 80.5%, respectively, as assessed by flow cytometry. A representative GFP expression is shown (Figure 1(a)). Based on these results, the transfection efficiency reached its maximum at a MOI of 200. After transfection, Bcl-2-ADSCs and vector-ADSCs were morphologically indistinguishable from ADSCs. Thus, the optimal MOI of 200 was chosen for subsequent experiments. Western blot analysis was performed to evaluate the expression of Bcl-2 protein in the genetically modified adipose-derived stem cells 24 hours, 72 hours, 7 d, and 21 d post-transduction. A significantly high expression of Bcl-2 was observed in Bcl-2-modified adipose-derived stem cells as early as 24 hours, which remained detectable on d 21 (Figure 1(b)). In contrast, weak expression of Bcl-2 was detected in vector-modified adipose-derived stem cells 24 hours post-transduction and in adipose-derived stem cells.

### Bcl-2-modified adipose-derived stem cells improved the retention of fat grafts

**Figure 2.**
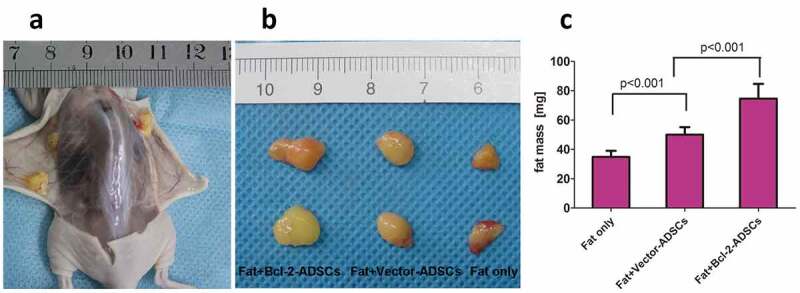
Retention of the fat grafts 6 weeks after transplantation. (a) Surviving fat grafts under the skin of a representative nude mouse. (b) Harvested fat grafts. (c) Mass of fat grafts was higher in the Fat + Bcl-2-modified adipose-derived stem cells group (Fat + Bcl-2-ADSCs) than in the Fat-only group, or than in the Fat + vector-modified adipose-derived stem cells group (Fat + Vector-ADSCs). The differences between any two groups were statistically significant.

At 6 weeks post-transplantation, the fat grafts were removed and weighed. Analysis of weight showed that the mean graft weight was 115% higher in the Fat + Bcl-2-modified adipose-derived stem cell group than in the Fat-only group and 49% higher than in the Fat + vector-modified adipose-derived stem cell group (Figure 2).

### Histological evaluation of fat grafts

**Figure 3.**
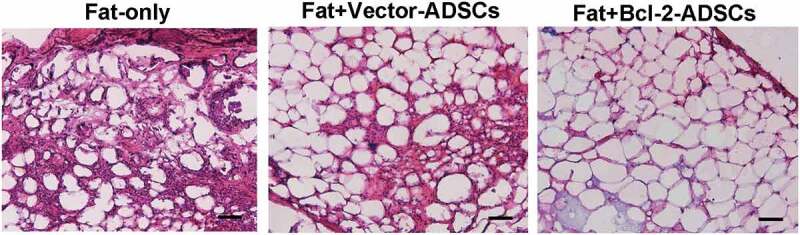
Histological features of transplanted fat tissues. Grafts from Fat + Bcl-2-modified adipose-derived stem cells group (Fat + Bcl-2-ADSCs) contained more adipocytes and lower levels of fat necrosis and fibrosis than those from the Fat-only group, or than those from Fat + vector-modified adipose-derived stem cells group (Fat + Vector-ADSCs). Scale bars = 50 µm.

Histological analyses showed that grafts from the Fat + Bcl-2-modified adipose-derived stem cell group had significantly more intact adipocytes and lower levels of fat necrosis and fibrosis than those from the Fat-only group or than those from Fat + vector-modified adipose-derived stem cell group at 6 weeks post-transplantation, while the grafts from the Fat-only group showed obvious infiltration with numerous cells of different types (Figure 3).

### Survival and location of Bcl-2-modified adipose-derived stem cells in fat grafts

**Figure 4.**
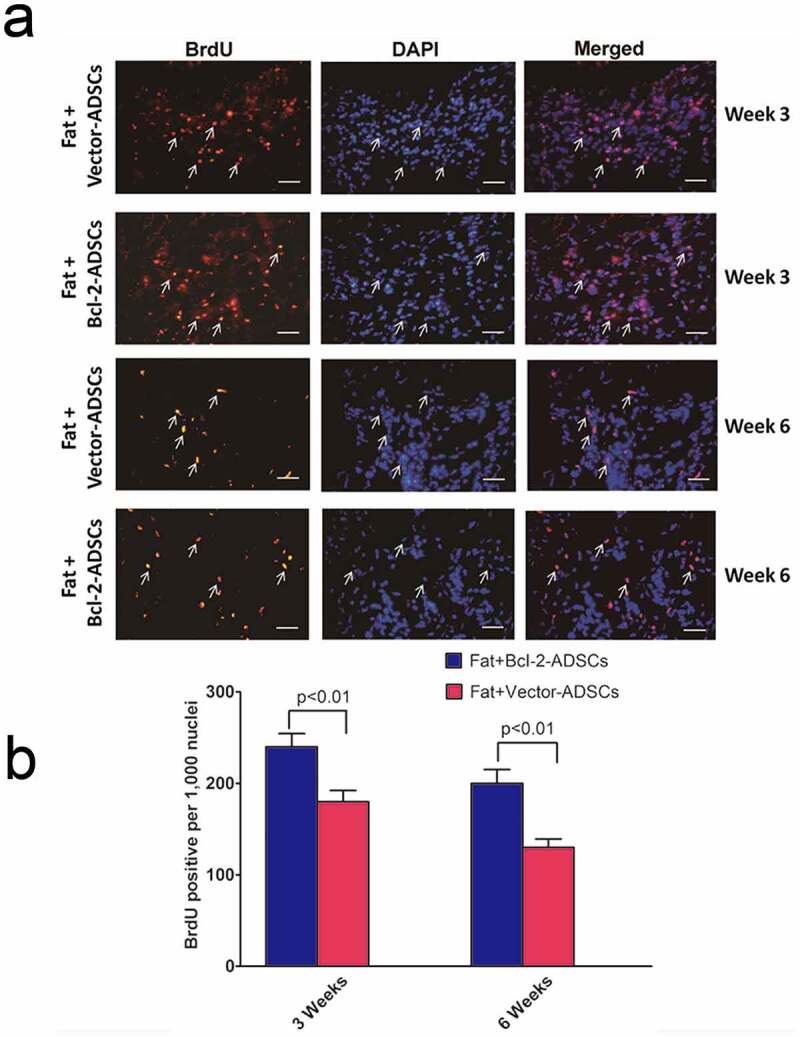
Bcl-2-modified adipose-derived stem cells in transplanted fats. (a) Representative images from immunostaining of transplanted adipose-derived stem cells in the Fat + vector-modified adipose-derived stem cells group (Fat + Vector-ADSCs) and in the Fat + Bcl-2-modified adipose-derived stem cells group (Fat + Bcl-2-ADSCs) 3 weeks and 6 weeks after transplantation. BrdU-labelled adipose-derived stem cells (brown) were clearly identified. (BrdU) (brown). (DAPI) (blue). Representative cell engraftment was detected (white arrows). (b) Quantitative assessment of surviving adipose-derived stem cells at 3 weeks, and 6 weeks. The number of surviving Bcl-2-modified adipose-derived stem cells was greater than vector-modified adipose-derived stem cells at each time point. Scale bars = 20 µm.

The assessment of vector-modified adipose-derived stem cells and Bcl-2-modified adipose-derived stem cell survival was carried out 3 weeks and 6 weeks after transplantation. Representative images of vector-ADSCs and Bcl-2-modified adipose-derived stem cells were demonstrated 3 and 6 weeks following transplantation (Figure 4(a)). BrdU-positive nuclei were detected by immunostaining (Figure 4(a)). The number of surviving Bcl-2-modified adipose-derived stem cells was significantly greater than vector-modified adipose-derived stem cells following transplantation (Figure 4(b)), with 33% enhancement of cell survival on week 3 (n = 8, *p* < 0.01), and 54% enhancement on week 6 (n = 8, *p* < 0.01).

**Figure 5.**
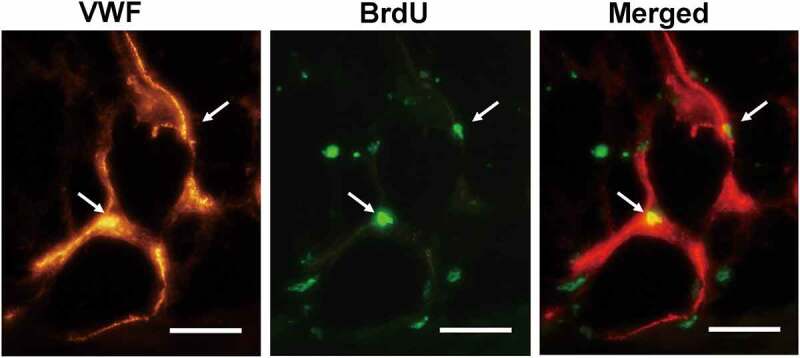
The angiogenic differentiation of Bcl-2-modified adipose-derived stem cells 3 weeks after transplantation. The vWF-positive vascular structure was red. The BrdU-positive Bcl-2-modified adipose-derived stem cells were green. In merged image of the vWF-positive vascular structure with the BrdU-positive Bcl-2-modified adipose-derived stem cells, arrows indicate the location of BrdU-positive and vWF-positive cells. Double immunofluorescence staining revealed Bcl-2-modified adipose-derived stem cells incorporated into the endothelial lining of neocapillary. Scale bars = 20 µm.

The assessment of Bcl-2-modified adipose-derived stem cell location was carried out 3 weeks after transplantation. Double immunofluorescence staining with BrdU and anti-vWF antibody in the Fat + Bcl-2-modified adipose-derived stem cell group revealed that some of the Bcl-2-modified adipose-derived stem cells (3.5% ± 1.3%) incorporated into the endothelial lining of neocapillary; the others were detected around the mature adipose tissue. Occasionally, blood vessels consisted of Bcl-2-modified adipose-derived stem cells (Figure 5).

### Capillary density

**Figure 6.**
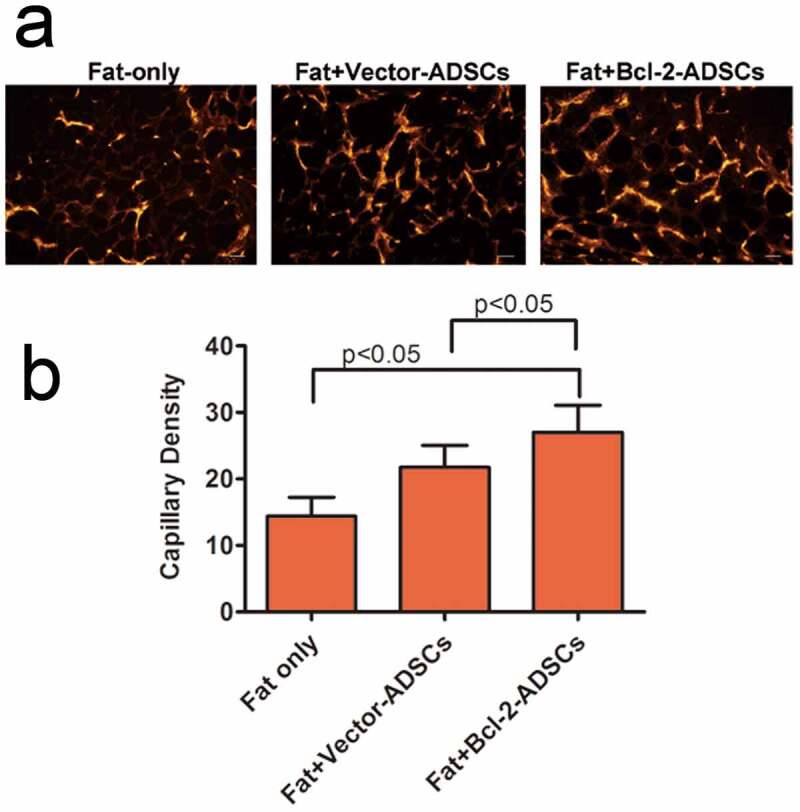
Bcl-2-modified adipose-derived stem cells enhanced angiogenesis in the transplanted fats. (a) Representative photomicrographs of the transplanted fats obtained after immunostaining for vWF antibody. (b) Quantitative capillary density data based on vWF immunostaining. Capillary density was much higher in the Fat + Bcl-2-modified adipose-derived stem cells group (Fat + Bcl-2-ADSCs) than in the Fat-only group, or Fat + vector-modified adipose-derived stem cells group (Fat + Vector-ADSCs). The differences between any two groups were statistically significant. Scale bars = 20 µm.

Capillary density in the grafts was determined based on vWF immunostaining 3 weeks after transplantation. The representative images are shown (Figure 6(a)). There was a significant increase in capillary density in Fat + vector-modified adipose-derived stem cells (21.8 ± 3.3 vessels per HPF) groups and Fat + Bcl-2-modified adipose-derived stem cells (27 ± 4.1 vessels per HPF) group when compared with Fat-only (27 ± 4.1 vessels per HPF) groups. The capillary density in Fat + Bcl-2-modified adipose-derived stem cell groups was 24% higher than that in Fat + vector-modified adipose-derived stem cell groups (n = 8, *p* < 0.05) or 60% higher than that in Fat-only groups (n = 8, *p* < 0.05) (Figure 6(b)).

## Discussion

In the past 10 y, the plasticity and therapeutic potential of adipose-derived stem cells have rapidly come into the focus of stem cell research [23]. Adipose-derived stem cells display similar properties to bone marrow-derived mesenchymal stem cells, which can be maintained for extended periods and proliferate rapidly with low levels of senescence and have the potential to differentiate into multiple lineages, such as bone, cartilage, adipose tissue, and muscle, when cultivated in appropriate culture medium [24,25]. Furthermore, adipose-derived stem cells can be harvested in significant numbers simply by easy liposuction from abundant subcutaneous fat depots without ethical concerns. Thus, the adipose-derived stem cells are increasingly expected to become a highly promising stem cell population thus far and a valuable tool in a wide range of cell-based therapies [26].

In cell-assisted lipotransfer, adipose-derived stem cells may have four functions [11,12]. First, some of vascular endothelial cells are differentiated from adipose-derived stem cells that may contribute to neoangiogenesis. Second, some adipose-derived stem cells such as adipose progenitor cells may play a role in the future turnover of adipocytes. Third, some adipose-derived stem cells can differentiate into mature adipocytes and partly constitute transplanted fat. Fourth, transplanted adipose-derived stem cells may release angiogenic growth factors, accelerating neoangiogenesis from the surrounding host tissue in a paracrine manner. However, previous reports have shown the poor viability of transplanted stem cells within the ischaemic conditions [15–17]. Therefore, protection of adipose-derived stem cells against apoptosis is critical in cell-assisted lipotransfer. A previous study has demonstrated that Bcl-2 modification can significantly reduce adipose-derived stem cell apoptosis [22,27] and enhance secretion of growth factors by adipose-derived stem cells under harsh conditions [28]. Protection of adipose-derived stem cells against apoptosis by Bcl-2 overexpression would lay a good foundation for improving the retention of fat graft.

In this study, transplantation of Bcl-2-modified adipose-derived stem cells mixed with fat resulted in improvement on the retention of human autologous fat graft and a substantial attenuation of necrosis and fibrosis in transplanted fat. The critical role Bcl-2 played in augmenting grafted adipose-derived stem cell survival under ischaemia was demonstrated in vivo. We detected some BrdU-positive Bcl-2-modified adipose-derived stem cells and vector-modified adipose-derived stem cells in the surviving fat tissue 3 or 6 weeks after transplantation. Although equivalent numbers of both Bcl-2-modified adipose-derived stem cells and vector-modified adipose-derived stem cells are mixed with fat grafts at the beginning of transplantation, Bcl-2 upregulation enhances the survival rate of adipose-derived stem cells in the fat grafts during prolonged ischaemic conditions, as suggested by the in vivo experiments (Figure 4). A previous finding from Fu et al. demonstrated that some adipose-derived stromal vascular fraction cells can spontaneously differentiate into adipocytes from d 7 after co-implantation with fat grafts [29]. Therefore, some of the Bcl-2-modified adipose-derived stem cells and vector-modified adipose-derived stem cells are believed to differentiate into mature adipocytes which may compensate for the early loss of transplanted fat. Some of the Bcl-2-modified adipose-derived stem cells and vector-modified adipose-derived stem cells survived as adipose progenitor cells may account for future turnover of adipocytes, this may contribute to the long-term retention of fat grafts. As the survival rate of Bcl-2-modified adipose-derived stem cells is much higher than that of vector-modified adipose-derived stem cells in fat grafts, the retention of fat transplantation with Bcl-2-modified adipose-derived stem cells is also higher than that of fat transplantation with vector-modified adipose-derived stem cells. A small number of BrdU-positive Bcl-2-modified adipose-derived stem cells were found in the vessel walls as identified by the staining of vWF (Figure 5), indicating that Bcl-2-modified adipose-derived stem cells could directly contribute to vessel formation by incorporation into vessel walls. However, this observation was shown at low frequency. Thus, it is possible that Bcl-2-modified adipose-derived stem cells may promote neovascularization in the fat grafts, to a very small extent, through differentiation into vascular endothelial cells. Our results agree with the earlier findings that adipose-derived stem cells may promote neovascularization in fat grafts mainly by paracrine mechanism [30–32]. We also found that the capillary density was higher in fat grafts with Bcl-2-modified adipose-derived stem cells. Taken together, these observations highlight the critical role for Bcl-2-modified adipose-derived stem cells in enhancing the retention of fat grafts and improving the quality of fat grafts.

Previous studies have reported that the function of adipose-derived stem cells in cell-assisted lipotransfer should be does-dependent [33,34]. Bcl-2 overexpression protects adipose-derived stem cells under ischaemic conditions; thus, fewer Bcl-2-modified adipose-derived stem cells could perform the same function as more adipose-derived stem cells alone. The efficacy of adipose-derived stem cells in cell-assisted lipotransfer could be enhanced largely by Bcl-2 modification, and the number of adipose-derived stem cells required would be reduced. This might be quite useful to some clinical cases with less available fat tissue. We could still achieve desirable retention of fat grafts with less adipose-derived stem cells by Bcl-2 modification.

In this experiment, we used adenovirus to mediate gene transduction. Here, instead of using lentivirus, the adenovirusbased transduction system is a transient expression system which allows protein expression in the transduced cells for a short period of time. This could minimize the possibility of tumorigenesis induced by Bcl-2 gene. Since passaged adipose-derived stem cells may display karyotypic abnormalities [35], long-term safety observation of this approach is necessary, such as the issue of neoplasia should be explored. It should be noted that it is at present not possible to use Bcl-2 modified adipose-derived stem cells in clinical settings due to some potential regulatory hurdles which will need to be overcome. A limitation with this study is the duration between graft implantation and explantation. A longer period (e.g. >12 weeks) would favour an observation about the final result of fat grafts. In addition, this study was performed in small animals lacking an intact immune system. The experiments need to be repeated in a larger animal model, more similar to human physiology.

## Data Availability

The datasets used and/or analysed during the present study are available from the corresponding author on reasonable request (https://doi.org/10.1080/21623945.2022.2107195).
